# Application of Electrochemical Techniques for Determining and Extracting Natural Product (EgCg) by the Synthesized Conductive Polymer Electrode (Ppy/Pan/rGO) Impregnated with Nano-Particles of TiO2

**DOI:** 10.1038/s41598-019-39952-2

**Published:** 2019-03-08

**Authors:** Fatemeh Ferdosian, Mehdi Ebadi, Ramin Z. Mehrabian, Maziar A. Golsefidi, Ali V. Moradi

**Affiliations:** Department of Chemistry, Faculty of Science, Gorgan Branch-slamic Azad University, Gorgan, Iran

## Abstract

The polypyrrole/polyaniline-based electrode (Ppy/Pan/TiO_2_/rGO) was fabricated via the electrophoretic deposition technique on fluorine-doped tin oxide (FTO)-coated glass. Physico-electrochemical adsorption/desorption of epigallocatechin gallate (EgCg) as an electroactive species was enhanced by the fabricated electrode compared to the electroless technique extraction using the same electrode. EgCg was electrochemically extracted using chronoamperometry by electrophoretically deposited Ppy/Pan/TiO_2_/rGO film. Isolated EgCg was qualified and quantified by the voltammetry and high-performance liquid chromatography (HPLC) techniques. It was found that the extracted EgCg values were 3.38 and 0.72 ppm from a 10 ppm prepared solution using the electrochemically and physically based techniques, respectively. Morphology/*elemental analysis* and crystal structure of the prepared electrodes were characterized by field emission scanning electron microscopy/energy-dispersive X-ray (FESEM/EDX) and X-ray diffraction (XRD), respectively. The conductivity of the fabricated electrode was investigated by electrochemical impedance spectroscopy (EIS) and was calculated as 1.124 S/cm for the electrophoretically deposited electrodes (EPD).

## Introduction

Flavonoids are products of secondary metabolism of plants and are found in a wide variety of herbs. Polyphenolic compounds are some of the most important biologically active substances in tea leaves. Green tea is a rich source of catechins that form up to 30% of the dried leaf weight. Catechin is the most commonly used food antioxidant that controls free radicals and plays an important role in preventing chronic diseases such as cancer^1^. Recent studies have shown that catechins of green tea can prevent some skin and liver cancers^2^. Catechins have anti-inflammatory, antioxidizing, antimicrobial, anticancer and antimutagenic properties^3^. They can also reduce the risk of lung cancer and stomach cancer. In the pharmaceutical industry, catechins are used in toothpaste, mouthwashes, and respirators for improving oral health. In addition, catechins play an important role in the food industry and food supplies. Catechins are well known as a supplement in nutritional drinks used for improving the health of the consumers^4^.

Catechins are colourless and dissolve both in water and polar organic solvents^1,5^. Catechins are divided into two groups: free catechins and stereoisomer catechins. Free catechins include catechin (C), galacacchine GC), epicatechin (EC), epigallocatechin (EGC), while EgCg, epicatechin gallate (ECG), galactic acid gallate (GCG) and catechin gallate (CG) are stereoisomer catechins. Stereoisomer catechins have a significant moisture content and a bitter taste, while free catechins are very harmful and have a slightly sweet taste^6^. Previous research has shown that most of the antioxidant activity of green tea is related to epigallocatechin gallate.

Identification, measurement and extraction of catechins as bioactive materials has attracted the attention of many researchers. Several methods are currently available for identifying and measuring tea catechins^1,7^. These methods can be used to determine the concentration, application, and purity of catechins in the final products. The identification and measurement of catechins have been greatly facilitated by chromatographic techniques such as HPLC and capillary electrophoresis (CE) using various detectors such as UV, electrochemical, and MS detectors for the analysis of individual catechins. Recently, an HPLC instrument accompanied with a set of appropriate detectors has been introduced and showed promising performance for the determination and separation of catechins^8^. The wavelengths of maximum absorbance for catechins have been reported at 210 and 269–280 nm^9,10^. Therefore, UV and diode array detectors have been widely used for the determination of individual catechins. The phenolic compounds and antioxidant capacity of green tea have been investigated by many researchers to find the optimal conditions for their extraction. The most important methods that have been used for the extraction of catechins are: solvent extraction, pure water and organic extraction^11,12^, extraction using microwave waves^13^, ultrasound-assisted extraction (UAE)^14^, ultrahigh-pressure extraction (UHPE)^15^, extraction by filtering^16^, extraction by molecular imprinted polymers extraction by polymeric agents^17^, supercritical water extraction (SWE)^18^, solid-phase extraction^19,20^ and supercritical water and fluid extraction (SWE-SFE)^21^. It was also reported that conductive polymers can be used for the separation of biocompatible active compounds.

Polyaniline and polypyrrole are among the most commonly used copolymers due to their environmental stability, electrical properties, absorption of organic dyes and their unique electrochemical properties that are useful for the fabrication of polymer electrodes^22^. Puanglek *et al*. have produced high-quality films via the electrochemical synthesis method for the separation of biological materials due to the control of the thickness and the possibility of doping during the synthesis of these films^23^. Owing to the importance of polymer electrodes, their refinement and optimization have attracted research interest. Some studies have reported that the efficiency and the properties of the polymers were enhanced when they were combined with nanostructured materials^23^. Recently, graphene oxide, carbon nanoparticles, cellulose nanoparticles, multi-wall carbon nanotubes and cadmium oxide nanoparticles have been used to modify polymeric electrodes^24^. In fact, these types of nanocomposites were used to enhance the separation properties of electroactive polymer electrodes.

Unfortunately, the aforementioned extraction methods have the following several drawbacks: use of dangerous organic solvents, risk of harmful environmental effects, low purity of extraction, low accuracy and repeatability, high cost and difficulties for industrial applications. In fact, all of these methods are far from green chemistry. This necessitates the development of simple, non-toxic, repeatable and inexpensive extraction methods. Therefore, it appears that the electrochemical method is a good candidate extraction approach due its high accuracy, low cost, friendly operation and rapid data collection. However, to the best of the authors’ knowledge, the electrochemical extraction of EgCg has been not reported to date. Electrochemical impedance spectroscopy (EIS) is a powerful electrochemical technique that has shown promising results in a wide range of electrochemical application such as the evaluation of electrode/electrolytes performance. This technique is based on physico-chemical phenomena. The impedance is calculated based on Ohm’s law:

Z = E/I where E is the electrical potential and I is the electrical current. The obtained EIS data are governed by the changes in the current/potential at the measured frequencies that determine the electrochemical behaviour of a typical cell (electrodes) when a three-electrode compartment is used.

In this study, to enhance the synergy and effectiveness of extraction methods, the electrochemically synthesized polymeric nanomaterial films were applied as a selective electrode for EgCg separation using the chronoamperometry technique. The purified EgCg was quantitatively and qualitatively verified by the voltammetry and HPLC techniques.

## Results and Discussion

Among of conductive polymers, PPy have been shown the vast potential to determine of biomolecules as biosensors. The low surface area was reported as a drawback of PPy^25^. To achieve the high surface area the PPy was conjugated with PAn.

To extract EgCg, a thin layer of Ppy/Pan/TiO_2_/rGO was prepared from dispersed particles in propanol solution using the electrophoretic-coating technique. Several variables were examined such as the distance between the anode and the cathode (2.5, 5, 7 and 9 cm), time (5, 10, 15 and 20 min), applied voltage (40, 50, 70 and 80 V) and the amount of the particles in the solution (0.1, 0.01 and 0.001 g in 100 ml). Notably, it was found that among of all the prepared thin layers with the different specified conditions, the layer synthesized under the conditions of 100 V, 20 min from solution, 0.001 g in 100 ml and the anode-cathode distance of 9 cm showed the best physico-electrochemical properties. Typically, the samples were characterized as follows.

### FTIR studies

**Figure 1:**
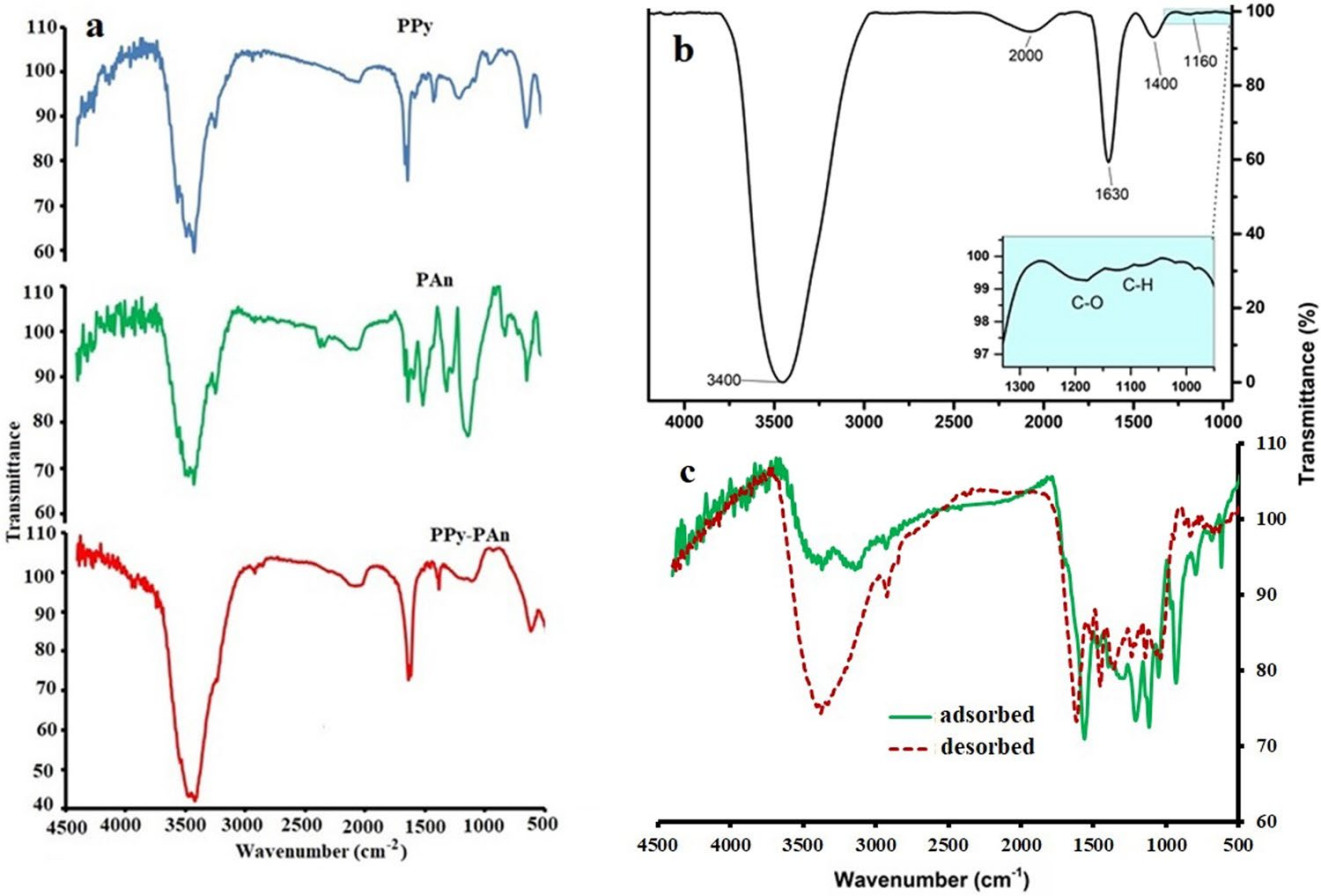
FT-IR spectra of: (**a**) Ppy, PAn and Ppy/PAn, (**b**) Ppy/Pan/TiO_2_/rGO; inset zoom area of graphene vibrational bending, (**c**) the spectrum of the adsorbed and desorbed EgCg on fabricated electrodes.

The spectra of PPy, PAn, PPy/PAn are compared in the range of 500–4500 cm^−1^ in Fig. 1a. In addition, the interaction of the initial materials to form the Ppy/Pan/TiO_2_/rGO composite as a final product was analysed further by FT-IR as depicted in Fig. 1b. The obtained characteristic peaks were as follows:

The band at the 1400 cm^−1^ corresponding to Ti-O-Ti vibration in the TiO_2_ structure. The peak intensity is proportional to the TiO_2_ content in the composite *(see experimental section)*. The bands observed at 1630 cm^−1^ and 3400 cm^−1^ are attributed to the bending vibration of the O-H of the chemically absorbed water and the stretching mode of adsorbed water, respectively, from the environment^26^. The high intensity of the O-H band can be attributed to the hydrophilic features of the final product that enable the absorption of more water compared to the other organic pollutants.

The characteristic bonds for graphene/graphene oxide in polymer composites were reported at 1200 cm^−1^ and 1580 cm^−1^ for the stretching modes of C-O and aromatic C=C, respectively^27^. The C=C bond signal was covered by the chemisorbed water and broad O-H vibration modes in the FT-IR spectrum, and in addition to the listed peaks, the high intensity of the broad bands at 1400 cm^−1^ and 1600 cm^−1^ could be ascribed to: i) strong interfacial interaction between graphene sheets and TiO_2_ nanoparticles that has a characteristic band at 1640 cm^−1^ for the C=O vibration that could be slightly shifted downfield to 1630 cm^−1^ for the Ti-O-C vibration mode due to the bonding with TiO_2_^27^.

ii) The C=C bond signals at 1580 cm^−1^ and 1460 cm^−1^ for the stretching mode in the Pan quinoid ring and for the stretching of the benzenoid ring, respectively, that are expected in the final product. The peak at 1627 cm^−1^ could be assigned to the skeletal vibrations of unoxidized graphitic domains.

Additionally, the slightly shifted frequencies of the observed bands could be attributed to the hydrogen bonding and *π-π* interaction between the graphene sheets and polyaniline in the composite product, providing further evidence for the successful formation of the composite^27^.

The characteristic bonds that indicate the presence of Pan and Ppy in the composite product were confirmed by the weak signal at 1160 cm^−1^ for the in-plane C-H bending in the Pan structure^28^. The shoulders at 1400 cm^−1^ and 2000 cm^−1^ were attributed to the stretching vibration of the C-N bond in the pyrrole ring in the final composite product^29^. Moreover, Fig. 1c has shown the adsorbed EgCg on the fabricated electrode, whereas; the spectrograph of desorbed EgCg was illustrated, as well.

### Morphology study

**Figure 2:**
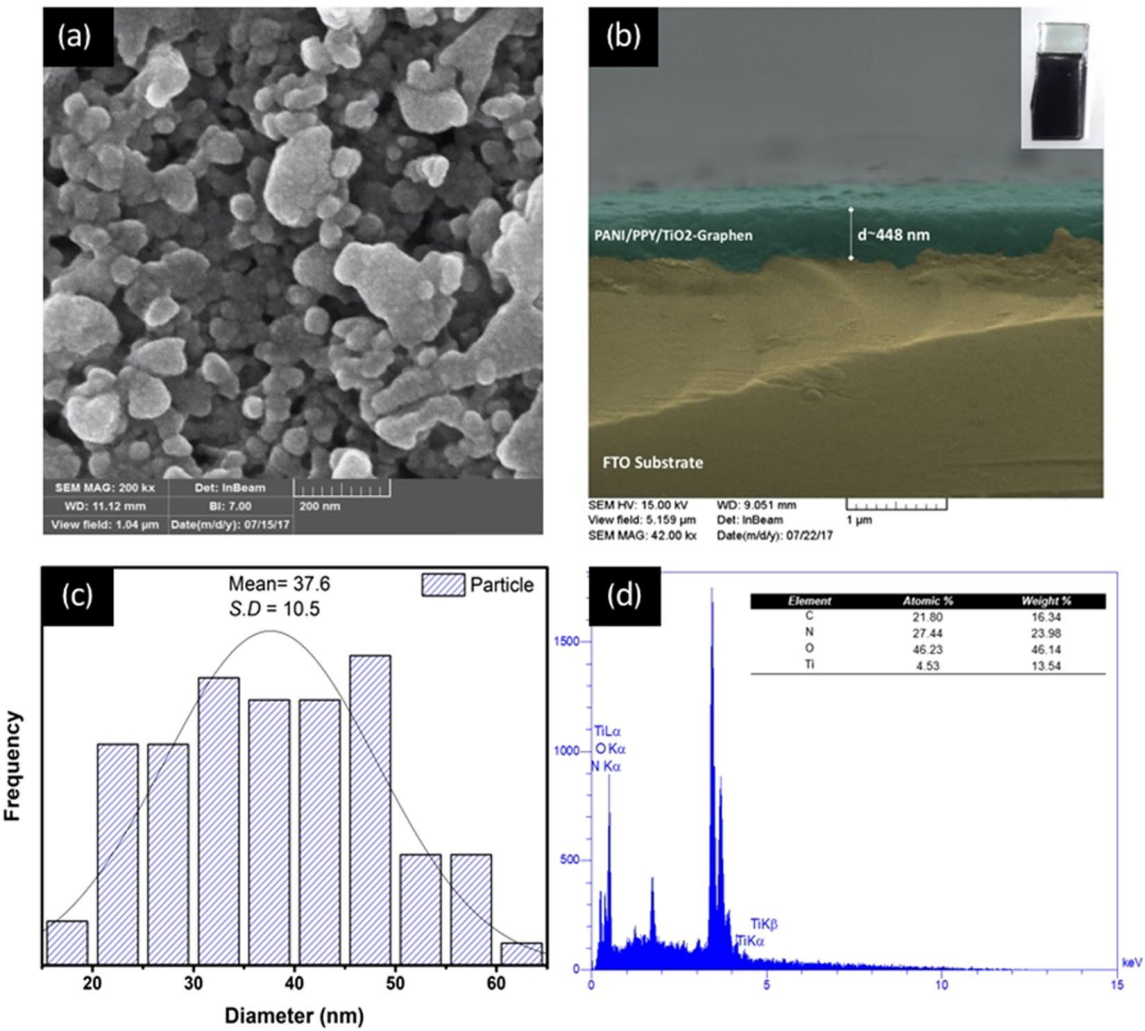
(**a**) FESEM image of Ppy/Pan/TiO_2_/rGO, (**b**) Cross-sectional SEM false-colored image of Ppy/Pan/TiO_2_/rGO sample composite on FTO substrate prepared by electrophoretic deposition (*inset electrode photograph*), (**c**) corresponding histogram of capsulated particles size distribution in (**a**)- Gaussian fit curve is shown in black, (**d**) EDX spectrum of sample composite with corresponding atomic and weight percentage of C, N, O and Ti.

FESEM images and corresponding elemental analyses are presented in Fig. 2. As shown in Fig. 2a, encapsulated particles were observed for the sample composite prepared by the electrophoretic deposition method. The average thickness of the composite film on the fluorine-doped tin oxide (FTO) substrate from the cross-sectional SEM micrograph (Fig. 2b) was calculated to be approximately 448 nm. The average diameters of the encapsulated particles were 37.6 ± 10.5 nm as obtained from the Gaussian fit of the histogram. EDX analysis confirmed the presence of C, N, O and Ti elements in the sample with the atomic percentages of 21.8, 27.44, 46.23 and 4.53%, respectively.

FESEM images and the corresponding elemental analyses are presented in Fig. 2. The morphology of the prepared particles was displayed by FESEM images as shown in Fig. 2a. Additionally, elemental analysis of the particles fixed on FTO was performed using EDX.

### XRD characterization

The bulk crystallinity and phase were characterized by X-ray powder diffraction (XRD, PHILIPS, PW1730, 40 kV/30 mA with Cu-K irradiation at = 1.5406 Å). The XRD scanning rate was 3.0 per minute in the range of 10° < 2θ < 99°.

**Figure 3:**
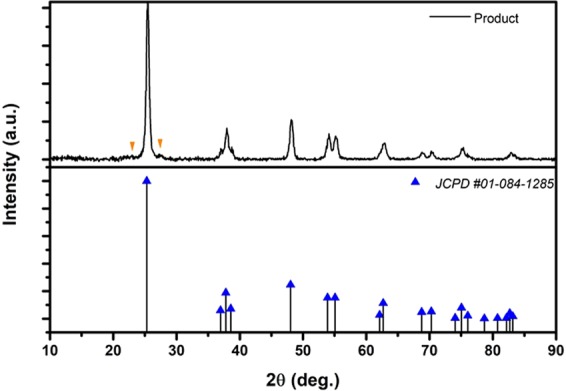
XRD spectra of Ppy/Pan/TiO_2_/rGO final product and relative TiO_2_ reference pattern.

The final Ppy/Pan/TiO_2_/rGO product with the diffraction peaks in the XRD pattern can be indexed to the tetragonal structure of TiO_2_ (anatase). As shown in Fig. 3, the peaks located at 2θ = 25.3°, 36.95°, 37.8°, 38.5°, 48.0°, 53.89°, 62.69°, 68.76°, 70.29°, 75.05°, 82.68° are related to the (101), (103), (004), (112), (200), (105), (204), (116), (220), (215) and (224) Miller indices, respectively, matching the reference pattern (JCPDS No. 01-084-1285). The average crystallite size of TiO_2_ was approximately 18 nm as calculated using the Scherrer’s formula^30^. The TiO_2_ peak at 25° dominates the Ppy and Pan characteristic peaks. For Pan, the peaks at 15.3°, 20.7° and 25.2° were reported for the crystal planes in the emeraldine salt form^31^. Ppy has a characteristic peak implying the highly oriented polymer chain or pyrrole intermolecular spacing that are centred approximately 24.6° and 25.4°, respectively^32^. Accordingly, in Fig. 3, two weak shoulders observed at approximately 23.5° and 27.3° were attributed to Ppy and Pan amorphous scattering, respectively.

### Determination of EgCg Using DC voltammetry

The current-potential (i-E) curves were recorded using a polarographic analyser. A rotating disc electrode (0.3 cm^2^) at a rate of 1000 rpm, Ag/AgCl, and a Pt-foil were used as the working, reference and auxiliary electrodes, respectively. A solution of EgCg (pH = 4) was adjusted with 0.1 M acetic acid^33^. The concentration of EgCg in analysed solutions was obtained by the standard addition method in the differential pulse (DP) mode while 50 μl of the studied solutions (rest and extracted from 10 ppm solutions) was diluted (400 times) and then was used as the electrolyte of the electrochemical cell. In addition, the electrolytes were buffered at the above-mentioned pH. Prior to recording each i-E curve, the solution of the cell was purged by a stream of pure nitrogen, for 5 min prior to the first recording and for 30 s after the addition of each aliquot. The analysed potentials ranged from 0.6 to −0.15 V, while the potential scan rate was 10 mV s^−1^, the pulse amplitude was 0.05 V, the pulse time was 0.04 s and the voltage step was 0.006 V. All of the experiments were performed in triplicate at room temperature.

Electroactivity of EgCg (10 ppm) was evaluated qualitative and quantitatively using the typical DC voltammetry technique. The DP method that is known for its capability to evaluate a trace amount of an electroactive solute was used to determine the EgCg content in the pH = 4 solution at room temperature^33^. To determine the oxidizing potential of EgCg, 50 μl of 10 ppm of the aqueous standard solution was added to the 20 ml voltammography cell in each calibrating step. The measurement was conducted in a three-electrode configuration with a rotating disc electrode as the working electrode, a platinum plate as the counter electrode and Ag/AgCl as the reference electrode. The potential was scanned in the range of +0.6 to −1.5 V.

**Figure 4:**
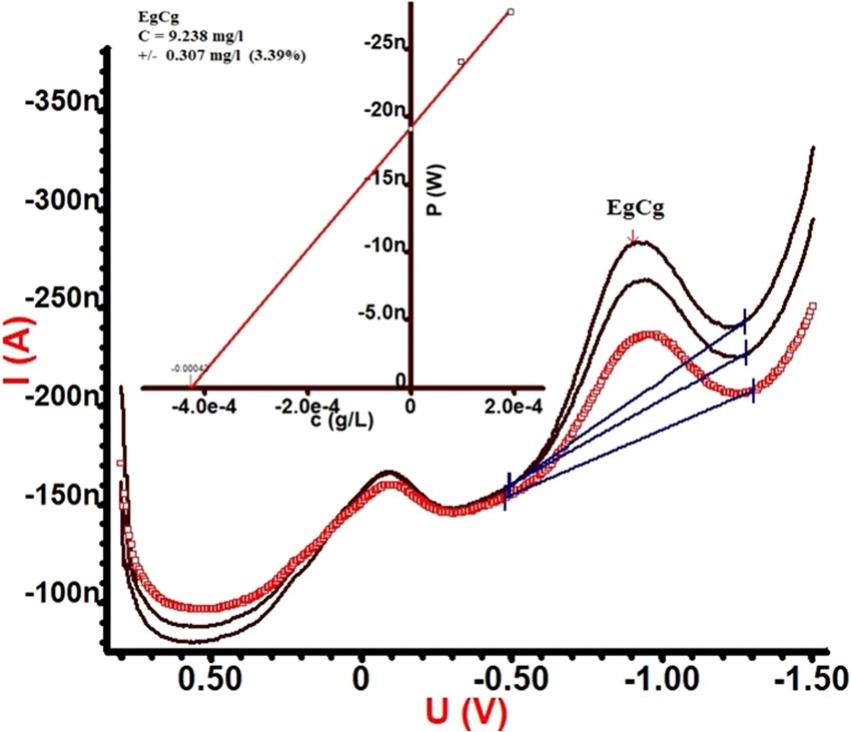
Voltammogram of EgCg (Inset calibration curve with linear fit).

As demonstrated in Fig. 4, two characteristic peaks appeared at 0.0 and −1.0 V (vs. Ag/AgCl). Upon increasing the appropriate amount of the standard solution in the sample, the intensity of these peaks started to increase, confirming that the EgCg in the environment is due to the increasing analyte concentration. The concentration of trace EgCg in the prepared sample (10 ppm) was calculated (9.238 ppm) by extrapolating the linear fit of the calibration curve (inset in Fig. 4). To the best of our knowledge, the reduction of EgCg has not been reported to date. By contrast, the oxidation of EgCg was examined by numerous scientists such as Zhang *et al*., and Masoum *et al*., using square wave voltammetry and differential pulse voltammograms, respectively^34,35^. The results obtained from DC voltammetry confirm the successful extraction by the method used in this study.

### EIS study of the electrochemical behaviour of the electrode

**Figure 5:**
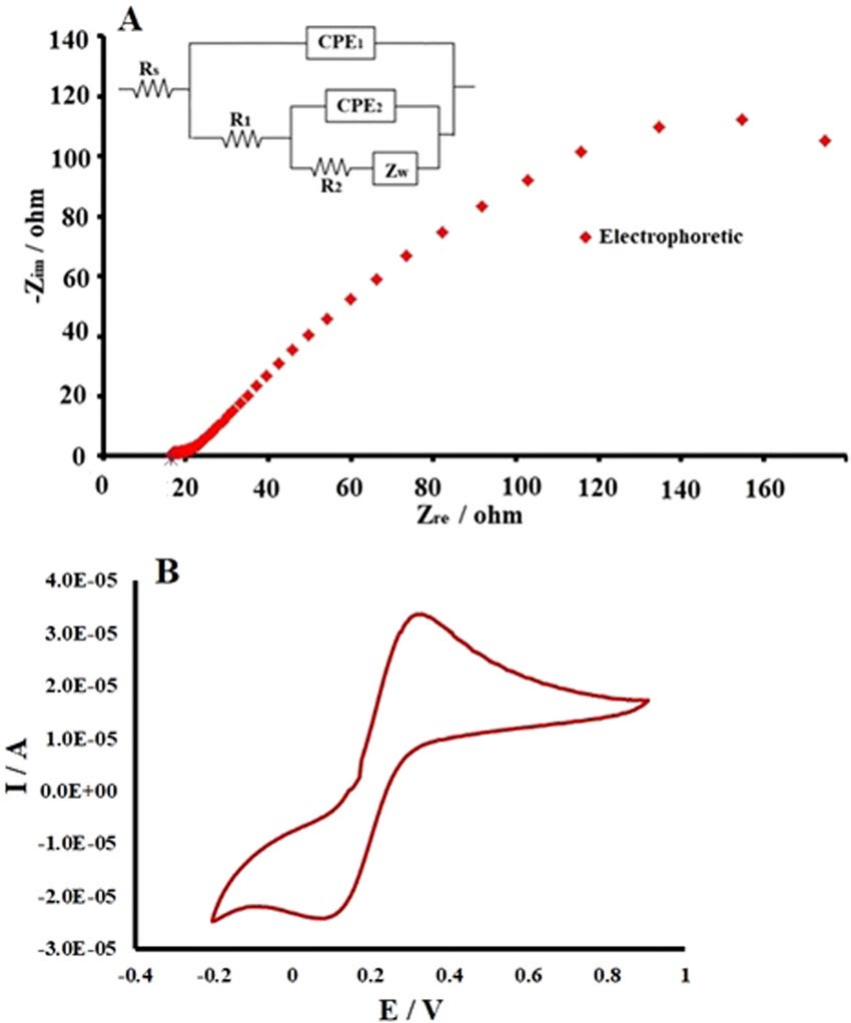
(**A**) The obtained electrochemical impedance spectra (EIS) for electrophoretic-deposited electrode at bias free potential *vs* calomel electrode using 0.1 M KCl solution, (**B**) cyclic voltammetry of potassium ferrocyanide (0.05 M) with the prepared electrodes.

Porous electrodes have shown many advantages for electrochemical approaches such as capacitance and adsorption. The greatest advantage of such electrodes is their large surface area. The amount of the species adsorbed on the electrodes surface depends on the conductivity, surface area of the electrodes and the applied voltage. The conductivity of electrochemical electrodes was studied by EIS, while the associated equivalent circuits of the electrodes was revealed from the excellent fitting of the EIS data, as shown in Fig. 5A.

A typical electrode was evaluated by the electrochemical impedance technique (Nyquist plot) using 0.1 M KCl. Figure 5 shows a curve for the electrophoretically obtained electrode in the abovementioned conditions. To study the electrochemical behaviour of the fabricated layer in more detail, the equivalent circuit[R_s_-(Q1│(R1-(Q2│R2-W))]with two relaxation time were used with the excellent fitting resulting in the chi square value of 10^−6^. The first (Q1R1) and second (Q2R2-W) loops of the equivalent circuit were ascribed to the fabricated layer and conducting surface layer of the FTO, respectively. Here R_s_ is the solution resistance, and R1 and R2 are the resistances of the deposited layer and the conducting layer of the FTO to the electron transfer. A constant phase element (CPE) was used instead of a Q element to represent the electrical double layer capacitance behaviour for both electrodes. Moreover, the Warburg (W) element appeared due to the diffusion of Cl^−^ ions to the fabricated layer^29^ and excellent agreement between experimental and simulation data was achieved for the best fit with the electrical analogue parameters listed in Table 1. The calculated conductivity of the electrophoretically deposited film was 1.124 S/cm^2^. Notably, the calculated conductivities are proportional to the electro-resistivity (arc of the curve of the Nyquist plot in Fig. 5A)

**Table 1:** Corresponding electrical parameters obtained by the equivalent circuit analogue simulation modelling for the electrophoretically deposited samples.

Electrode	R_s_ (Ω)	R_1_ (Ω)	Q_1_ (Y_o_) (mMho)	n	Q_2_ (Y_o_) (mMho)	n	R_2_ (Ω)	W (mMho)	S/cm
Electrophoretic	15.7	8.3	450.2	0.57	673.24	0.63	888.9	15.86	1.124

Furthermore, the conductivity of the synthesized electrode was investigated using the cyclic voltammetry technique in 0.05 M potassium ferrocyanide as shown in Fig. 5B. The oxidation reduction peaks of potassium ferrocyanide were prominently displayed.

### Chronoamperometry measurement

Chronoamperometry measurements were conducted to determine the adsorption/desorption content of EgCg. The Cottrell relation was used for this purpose:

1 $$I=\frac{nFAC{D}^{0.5}}{{\pi }^{0.5}{t}^{0.5}}$$

where I is the current (A), n is the number of exchanged electrons, F is the Faraday constant (C), A is the electrode surface area (cm^2^), C is the analyte concentration (M), D is the diffusion coefficient (cm^2^/s) and t is the time (s)^36^.

**Figure 6:**
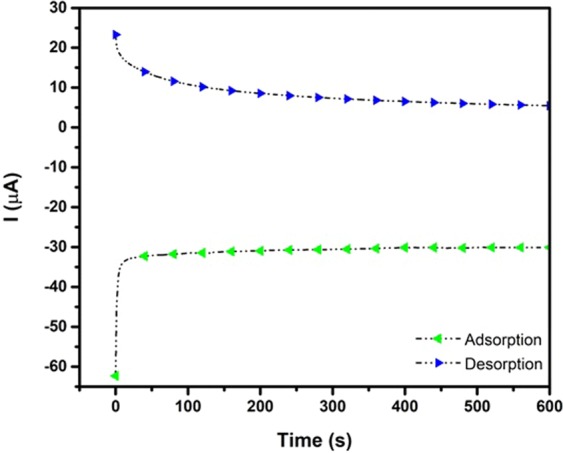
(**a**) Chronoamperometry extraction of EgCg (10 ppm) solution by Ppy/Pan/TiO_2_/rGO electrode. Adsorption (*blue*) and desorption (*green*) process.

Adsorption/desorption measurements were conducted for Ppy/Pan/Tio_2_/rGO synthesized *via* the electrophoretic deposition method. The desorption time was 3600 s at −1.3 V (*vs*. Ag/AgCl) and 1800 s at +1.0 V, as shown in Fig. 6. The average values for the adsorption and desorption process were obtained from three measurements in the tests with the durations of 1 h and 20 min, respectively. The residual solution was kept for further analysis by HPLC.

**Figure 7:**
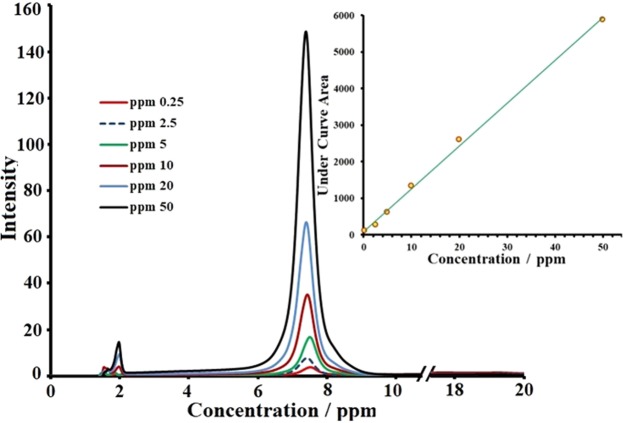
HPLC spectrums of the solutions with different concentration of EgCg which accompanied by associated calibration curve.

To qualify and quantify the EgCg in extracted and rest media, a series of solutions with different concentrations (0.25, 2.5, 5, 10, 20 and 50 ppm) were prepared and tested using an HPLC analyser. Figure 7 shows that all of the peaks related to EgCg appeared at the same retention time (7.85 min). Interestingly, it was observed that the peak area increased with increasing solution concentration. The obtained calibration curve is also shown in Fig. 7. The straight line was obtained from calculated peak areas.

**Figure 8:**
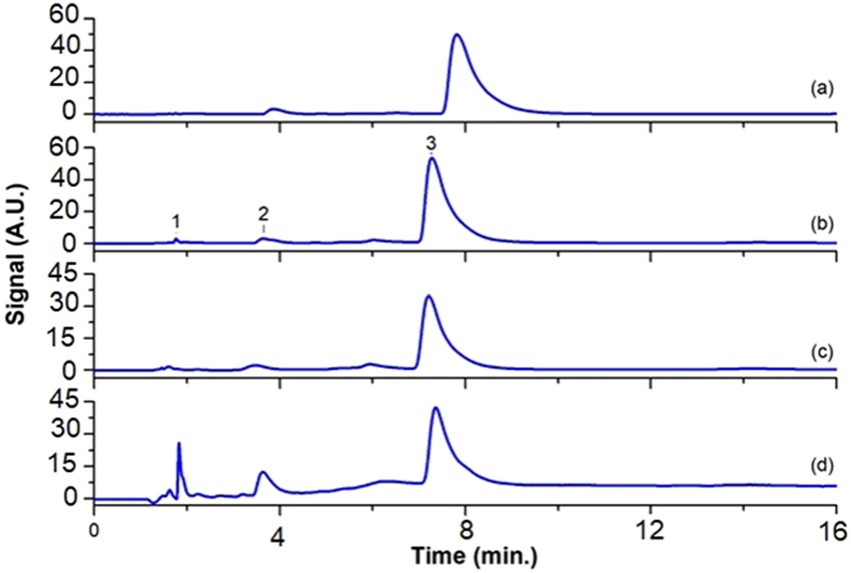
The HPLC chromatogram (**a**) ST-10ppm, (**b**) the rest solution after physically adsorption, (**c**) the solution obtained after electrochemically desorption, (**d**) the rest solution after electrochemically desorption.

Chromatographic peaks in the samples were identified by comparing their retention time and UV spectrum with those of the reference standards. Working standard solutions (10 ppm) were injected into the HPLC, and the peak area responses were obtained (Fig. 8a).

Adsorption/desorption measurements of EgCg with electrophoretically synthesized Ppy/Pan/TiO_2_/rGO were investigated by HPLC. In this method, standard solution (200 µl, 10 ppm) and adsorbed solution residues (200 µl) and of desorbed solution (200 µl, obtained from chronoamperometry technique) were injected into the instrument as the samples. A strong signal for the standard solution was observed at 7.85 minute at 280 nm. Figure 8b displays the EgCg amount in the rest solution after physical extraction for 24 h in the electroless condition, while Fig. 8c,d show the EgCg-associated peaks for the desorbed and residue solutions, respectively

According to Fig. 8, the adsorption and desorption amounts of EgCg (obtained by chronoamperometry) from solution containing 10 ppm of the electroactive nanocomposite (EgCg) at the above-mentioned conditions were 5.03 ppm and 3.38 ppm, respectively. By contrast, for the electrochemical extraction technique, the adsorption/desorption values of EgCg were determined as 0.79 and 0.72 ppm by same electrode at the electroless conditions, respectively. Thus, a quite remarkable result was obtained that the natural electroactive species (such as EgCg) could be efficiently extracted by the electroconductive modified (Ppy/Pan/TiO_2_/rGO) electrodes. The obtained results are presented in Tables 2 and 3. The reduction potential of EgCg (Fig. 4) leads to the choice of an appropriate potential for successful adsorption/desorption using the chronoamperometry technique. The successful electrochemical method for qualitative/quantitative detection of EgCg was confirmed by HPLC results.

**Table 2:** Results for the extraction process from the solutions containing 10 ppm via the chronoamperometric adsorption/desorption (after 3 times replications).

Sample	Content (mg/l)	Time (s)	Voltage (V)	HPLC	
				Adsorption (ppm)	Desorption (ppm)
Ppy/Pan/TiO_2_/rGO	10	1200	−1.3	5.03	—
Ppy/Pan/TiO_2_/rGO	10	600	+1	—	3.38

**Table 3:** Results for the extraction process from the solutions containing 10 ppm via the electroless adsorption/desorption.

Sample	Content (mg)	Time (h)	HPLC	
			Absorption (ppm)	Desorption (ppm)
Ppy/Pan/TiO_2_/rGO	1	24	0.79	—
Ppy/Pan/TiO_2_/rGO	1	12	—	0.72

## Conclusion

A thin film of Ppy/Pan/TiO_2_/rGO was successfully coated on FTO glass using the electrophoretic-deposition technique. The deposited film was characterized by FTIR, EIS, FESEM/EDX and XRD. From EIS results, it was calculated that the conductivity of electrophoretic-deposited film was 1.124 S/cm. Voltammetry measurements showed that EgCg was an electroactive species and was qualitatively detected at −1 V, while the concentration of the 10 ppm EgCg solution was evaluated as 9.238. The extraction of EgCg was 3.38 ppm with the electrophoretically deposited film from the EgCg solution (10 ppm) whereas only 0.72 ppm was obtained by physical extraction with the same electrode from the same solution. The extracted EgCg was analysed by HPLC and voltammetry techniques, obtaining comparable results. This study has shown that the extraction of natural electroactive species can be successfully carried out using electrochemical techniques.

## Methods

### Reagents and materials

Except for HCl (37%), propanol (99%) and sodium dodecyl sulphate (98.5%) which were purchased from Sigma-Aldrich Co., all of the other chemicals used in this work (analytical grade) were obtained from Merck Co. Moreover, conductive fluorine-doped tin oxide glasses (FTO) were obtained from Pilkington (FTO TEC 18 Ω/cm^2^).

### Polymerization of nanocomposite particles (Ppy/Pan/TiO_2_/rGO)

Prior to any attempt for the polymerization process, both pyrrol and aniline were distilled to prepare a solution containing a high concentration of the monomers. First, for each 155 mM monomer solution, the pH was adjusted to 4 using HCl (1.5 M)^37^ and the solution was stirred for 1 h. The synthesis of the mixture-copolymer-based particles was initialized once TiO_2_ nanotubes (3% w/w) and graphene oxide (0.2% w/w) were added to the mentioned solution with 310 mM of ammonium persulphate that was added dropwise to the above solution at −5 °C and pH = 4 for 24 h. The synthesized nanoparticles were filtered and rinsed by distilled water and then were dried at 50 °C^25^.

Electrophoretic coating of the synthesized particles as a thin film on the FTO glasses were performed as follows: nanoparticles were dispersed (1 h) in 70% propanol in an ultrasonic bath. FTO surface was degreased with acetone and ethanol solvents, respectively. The surface of FTO was electrochemically coated by the nanocomposite particles (Ppy/Pan/TiO_2_/rGO) when it was connected to the cathode clips and a graphite rod (equal surface) used as the anode and these were immersed in the 70% propanol solution containing the abovementioned particles. Electrical potential was applied with a DC digital supply. The following synthesis parameters were used: time (5, 10, 15 and 20 min), applied voltages (40, 50, 70 and 80 V), amount of the particles in the solution (0.1, 0.01 and 0.001 g in 100 ml) and distance between the anode and cathode (2.5, 5, 7 and 9 cm).

### Characterization of coated films on FTO

The uniformly coated films on the FTO were characterized by FTIR, XRD, FESEM/EDX and EIS to evaluate the functional groups, crystal structure, visual morphology/elemental analysis and electrical conductivity of the coated layers, respectively.

### Using prepared films for extraction of EgCg

EgCg was extracted from solution containing 10 ppm (10 ml) by the physical and electrochemical adsorption/desorption techniques. i) Physically adsorption of EgCg was performed when the prepared surface was immersed in the 10 ppm solution for 24 h and EgCg was desorbed in distilled water (10 ml) for 12 h. A series of solutions with different pH were examined and it was found that pH 4 was optimal for the desorption step. ii) Electrochemical adsorption/desorption was performed by chronoamperometry applying −1.3 V (for 1200 sec)/+1 V (for 600 sec) using a PS/GS Autolab instrument. It must be mentioned that both adsorption/desorption and even EIS were carried out in a three-electrodes compartment. The conductivity of the films was analysed by EIS and cyclic voltammetry (CV) techniques in 1 M KCl at free bias potential and frequency range (100 kHz–0.1 Hz) and in 0.05 M potassium ferrocyanide (at −0.2–1 V), respectively. For all electrochemical aforementioned voltammetry, chronoamperometry and EIS techniques, Ag/AgCl and Pt plane (1 cm^2^) were used as the reference and counter electrodes, respectively.

### Determination of extracted EgCg

Extracted and rest solutions (20 μl) were analysed by differential pulse voltammetry (Computrace 797 VA) in the following conditions: Pt, Ag/AgCl and glassy carbon rotating disc electrode (0.3 cm^2^) with 1000 rpm were used as the counter, reference and working electrodes, respectively. All electrodes were immersed in the EgCg solution (10 ppm) while the potential range (+0.8 to −1.5 V) was scanned at a rate of 10 mV/sec in room temperature. 20 μl of solution 10 ppm was injected to the HPLC (agilent 1200): column C18 (15 cm), ultraviolet detector and mobile phase consisted acetonitrile solution (8%) + orthophosphoric acid 92% (0. 1% cons.) at flow rate 1 ml min^−1^.

## Data Availability

All data generated or analyzed during this study are included in this published article.
